# Regular Breakfast and Blood Lead Levels among Preschool Children

**DOI:** 10.1186/1476-069X-10-28

**Published:** 2011-04-01

**Authors:** Jianghong Liu, Linda McCauley, Charlene Compher, Chonghuai Yan, Xiaoming Shen, Herbert Needleman, Jennifer A Pinto-Martin

**Affiliations:** 1 University of Pennsylvania, School of Nursing, 418 Curie Blvd., Room 426, Claire M. Fagin Hall, Philadelphia, Pennsylvania 19104-6096, USA; 2 Emory University, Nell Hodgson School of Nursing, 1520 Clifton Rd NE # 402, Atlanta, GA 30322-4201, USA; 3 Shanghai Jiaotong University School of Medicine, 1665 Kong Jiang Road, Shanghai 200092, China; 4 University of Pittsburgh, UPMC, 200 Lothrop St., Pittsburgh, PA 15213-2582, USA

**Keywords:** lead exposure lead poisoning nutrition diet nutrients breakfast

## Abstract

**Background:**

Previous studies have shown that fasting increases lead absorption in the gastrointestinal tract of adults. Regular meals/snacks are recommended as a nutritional intervention for lead poisoning in children, but epidemiological evidence of links between fasting and blood lead levels (B-Pb) is rare. The purpose of this study was to examine the association between eating a regular breakfast and B-Pb among children using data from the China Jintan Child Cohort Study.

**Methods:**

Parents completed a questionnaire regarding children's breakfast-eating habit (regular or not), demographics, and food frequency. Whole blood samples were collected from 1,344 children for the measurements of B-Pb and micronutrients (iron, copper, zinc, calcium, and magnesium). B-Pb and other measures were compared between children with and without regular breakfast. Linear regression modeling was used to evaluate the association between regular breakfast and log-transformed B-Pb. The association between regular breakfast and risk of lead poisoning (B-Pb≥10 μg/dL) was examined using logistic regression modeling.

**Results:**

Median B-Pb among children who ate breakfast regularly and those who did not eat breakfast regularly were 6.1 μg/dL and 7.2 μg/dL, respectively. Eating breakfast was also associated with greater zinc blood levels. Adjusting for other relevant factors, the linear regression model revealed that eating breakfast regularly was significantly associated with lower B-Pb (*beta *= -0.10 units of log-transformed B-Pb compared with children who did not eat breakfast regularly, *p *= 0.02).

**Conclusion:**

The present study provides some initial human data supporting the notion that eating a regular breakfast might reduce B-Pb in young children. To our knowledge, this is the first human study exploring the association between breakfast frequency and B-Pb in young children.

## Background

Lead exposure, even at low levels, has been associated with demonstrable deficits in cognition and intelligence [1-6]. The extent and rate of lead absorption are influenced by physiologic factors including age, fasting state, and calcium and iron status [7,8]. Studies have shown that the absorption of lead in the gastrointestinal tract is more rapid in subjects who are in a fasting state [7,9,10]. In adults, the absorption rate of lead ranges from 60-80% among fasting subjects, about 10 times higher than when ingested with food [7,9]. Micronutrients are thought to interact with lead in the body and previous epidemiologic studies have shown that dietary micronutrient intakes influence B-Pb [8,11,12].

Ingested lead is absorbed at higher rates (about 40-50%) in infants and children than in adults [7,13]. Given the observed impact of fasting on lead absorption in adults, eating frequent and regular meals/snacks is recommended by health authorities for preventing lead poisoning in children [14]. In previous epidemiologic studies, B-Pb in children were positively associated with higher intakes of micronutrients such as calcium [15,16]and iron [17,18] and total calorie[11,12]. To our knowledge, however, there have been no prior epidemiology studies evaluating the link between breakfast consumption and B-Pb in children. After the phase-out of lead in gasoline in the year 2000, the mean blood lead level of children aged 0-14 years in China was 8.1 μg/dL, with 23.9% of children's having B-Pb greater than 10 μg/dL, the level established in 1991 as an indication of concern by the US Centers for Disease Control and Prevention (CDC) and adopted by the Ministry of Health of China in 2006 [19]. In this study, we aim to test whether regular breakfast consumption predicts B-Pb or risk of lead poisoning in Chinese preschool. Study subjects are the part of the China Jintan Child Cohort Study [20].

## Methods

### Data collection

Institutional review board approval was obtained from the University of Pennsylvania and the ethical committee for research at Jintan Hospital in Jintan, China. Four pre-schools (Jianshe, Huacheng, Xuebu, and Huashan) were chosen to represent city, suburban, and rural areas, respectively. Between Fall 2004 and Spring 2005, 1,757 male and female children (aged 3 to 5 years) attending the four preschools were invited to participate in this study. Parents of 1,656 (94%) children signed informed consents to approve their child's participation. A questionnaire containing questions regarding breakfast-eating habit (regular or not), demographics (gender, age, parental educations, parental occupations, and passive smoking exposure), and eating behaviors was completed by a parent. Eating breakfast regularly was defined as eating breakfast at least five days per week. We also conducted three days of 24-hours dietary recall in a subset of study population. The forms were given to the parents of children and collected by the research assistants on the following day. The intake of lunch for two weekdays at school was observed by research assistants (MPH students from South Eastern University). These dietary records were analyzed using the self-developed computerized nutrients assessment system which was similar to Food Processor (Food Processor; ESHA Research, Salem, OR).

Blood specimens of 1,344 children were collected by trained pediatric nurses using a strict research protocol to avoid lead contamination. We were not able to successfully collect blood samples from some children due to a variety of reasons (e.g. parents did not allow blood drawn, blood specimen not viable), but there were no differences in demographics between children with (n = 1344) and without blood samples (n = 312). Samples were frozen and shipped to the Research Center for Environmental Medicine of Children at Shanghai Jiaotong University for the analysis of lead and micronutrients (iron [Fe], copper [Cu], zinc [Zn], calcium [Ca], and magnesium [Mg]) concentrations in blood, using graphite furnace atomic absorption spectrophotometer [21,22]. This laboratory has participated successfully in a CDC-administered quality-control program (Blood Lead Proficiency Testing Program) for the measurement of lead in whole blood. Analysis of each specimen was conducted using a replication procedure, and the mean of the repeated measurements was taken as the final measure. Blood lead reference materials for quality control (QC) were provided by Kaulson Laboratories, New Jersey. QC samples were inserted blindly among the study samples (one QC sample in every 10 study samples. Limit of detection (LOD) of B-Pb was 1.8 ug/dL and half of LOD was imputed for 3 (0.2%) samples under LOD, which was among multiple runs (mean LOD).

### Statistical analysis

Demographic characteristics of children and parents/grandparents, nutrient and blood lead levels were compared between children eating breakfast regularly and those not eating breakfast regularly (defined as breakfast consumption at least five mornings per week), using chi-square test for categorical variables and t-test or Wilcoxon rank sum test for continuous variables (means or medians). Multivariate stepwise linear regression models of the dependent variable log-transformed B-Pb were fitted including breakfast frequency (regular vs. irregular) and other covariates such as demographic characteristics (children's age, gender, parental educations and occupations, grandparental educations), children's nutrient levels in blood, passive smoking, breast feeding history, and breakfast type (rice/noodle, meat mostly, and fruit mostly). A final model was obtained by applying a backwards elimination procedure using variables correlated with B-Pb with a p-value < 0.10. Logistic regression analysis was also performed to examine the associations between breakfast frequency and lead poisoning (B-Pb≥10 μg/dL). All analyses were done in SAS 9.2 (SAS Institute, Cary, NC).

## Results

Demographic variables for the entire sample and by regular breakfast eating groups are in Table 1. Children eating breakfast regularly had a lower median B-Pb (6.1 vs. 7.2 μg/dL, Wilcoxon test without controlling for covariates, P < 0.01) compared to those without regular breakfast consumption. Proportions of lead poisoning (B-Pb > = 10 μg/dL) were 8% among children with regular breakfast eating habit and 10% among those not eating breakfast regularly, but this difference is not statistically significant (chi-square test). Sixty-four percent of children eating breakfast regularly and 68% of children without regular breakfast had moderately high B-Pb (in the range of 5 and 10 μg/dL). There were no differences in gender and age distributions between children eating breakfast regularly and those not. Six percent (40/679) of boys and seven percent (47/564) of girls did not eat breakfast regularly. Children eating breakfast regularly tended to live in urban (41%) and suburban (39%) areas. Grandparent and parent characteristics were correlated with breakfast frequency. Grandparents of children who eat breakfast regularly were more likely to be educated. Parents of children who eat breakfast regularly were more likely to have higher education (30% of fathers and 20% of mothers finished college) and to be technicians and professional workers (39% of fathers and 30% mothers), as compared to those who did not regularly eat breakfast (13% of fathers and 8% of mothers finished college). There were no statistical differences in micronutrient levels other than higher Zinc (Zn) levels among children who ate breakfast regularly.

**Table 1:** Basic and nutritional factors among children

	Regular breakfast?			
**Characteristics**	**No**	**Yes**	***P *value**^‡^	**Total**

Blood lead level (μg/dL)				

Median (P5,P95) ^+^	7.2 (2.9, 12.8)	6.1 (2.7,10.9)	0.008	6.2 (2.8,10.9)

> = 10 μg/dL, n(%)	8 (10.4)	92 (7.9)	0.43	100 (8.0)

Gender			0.63	

Boy	40 (51.9)	639 (54.8)		679 (54.6)

Girl	37 (48.1)	527 (45.2)		564 (45.4)

Age			0.08	

3	23 (31.2)	314 (26.9)		337 (27.1)

4	18 (23.4)	419 (35.9)		438 (35.2)

5	35 (45.4)	433 (37.2)		468 (37.7)

School Area			0.03	

Urban	25 (32.5)	478 (41.0)		503 (40.4)

Suburban	27 (35.1)	454 (38.9)		481 (38.7)

Rural	25 (32.5)	234 (20.1)		259 (20.8)

Father's education			0.0002	

Less than high school	46 (59.8)	438 (37.6)		484 (38.9)

High school	21 (27.3)	378 (32.4)		399 (32.1)

College & University	10 (13.0)	350 (30.0)		360 (29.0)

Mother's education			0.0003	

Less than high school	56 (72.3)	575 (49.3)		631 (50.8)

High school	15 (19.5)	351 (30.1)		366 (29.4)

College & University	6 (7.8)	240 (20.6)		246 (19.8)

Father's occupation			0.005	

Unemployed	3 (3.9)	46 (3.9)		49 (3.9)

General labor	50 (64.9)	595 (51.0)		645 (51.9)

Technician/Professional	15 (19.5)	450 (38.6)		465 (37.5)

Other	9 (11.7)	75 (6.4)		84 (6.8)

Mother's occupation			0.04	

Unemployed	3 (3.9)	67 (5.8)		70 (5.6)

General labor	37 (48.1)	475 (40.7)		512 (41.2)

Technician/Professional	12 (15.6)	344 (29.5)		356 (28.6)

Other	25 (32.5)	280 (24.0)		305 (24.5)

Parents divorces or separated			0.89	

Yes	3 (4.6)	46 (4.3)		49 (4.3)

No	62(95.4)	1030 (95.7)		1092 (95.7)

Grandfather's education			0.007	

None	17 (22.1)	139 (11.9)		156 (12.6)

< = 9 years	29 (37.7)	591 (50.7)		620 (49.9)

> 9 years	31 (40.3)	436 (37.4)		467 (37.5)

Grandmother's education			0.01	

None	22 (28.6)	209 (17.9)		231 (18.6)

< = 9 years	19 (24.7)	483 (41.4)		502 (40.3)

> 9 years	36 (46.7)	474 (40.7)		512 (41.1)

Passive smoking			0.34	

Yes	46 (59.8)	632 (54.2)		678 (54.5)

No	31 (40.2)	534 (45.8)		565 (45.5)

Feeding method (during the first year)			0.42	

Breast feeding	63 (82.9)	889 (76.5)		952 (76.9)

Bottle (formula) feeding	5 (6.6)	92 (7.9)		97 (7.8)

Mixed	8 (10.5)	181 (15.6)		189 (16.3)

Breakfast type			0.12	

Noodle/Rice	50 (78.1)	760 (69.1)		810 (69.9)

Mixed	14 (21.9)	340 (30.9)		354 (30.1)

Calcium				

Mean ± SD	1.66 ± 0.21	1.64 ± 0.19	0.45	1.64 ± 0.24

Number of deficiency (%)	31 (40.3)	452 (38.8)	0.79	483 (38.9)

Cu				

Mean ± SD	26.96 ± 7.10	27.03 ± 6.80	0.83	27.05 ± 6.64

Number of deficiency (%)	1 (1.3)	6 (0.5)	0.34	7 (0.6)

Fe				

Mean ± SD	8.12 ± 0.87	8.13 ± 0.84	0.68	8.13 ± 0.83

Number of deficiency (%)	21 (27.3)	280 (24.0)	0.51	301 (24.2)

Mg				

Mean ± SD	1.50 ± 0.17	1.47 ± 0.16	0.10	1.47 ± 0.16

Number of deficiency (%)	0	4 (0.3)	0.61	4 (0.3)

Zn**^,+^				

Mean ± SD	78.25 ± 12.51	82.70 ± 13.27	0.008	82.46 ± 13.40

Number of deficiency (%)	41 (53.2)	436 (37.4)	0.006	477 (38.3)

^‡^, P value, comparison between children with and without regular breakfast; ^+^, P < 0.01, Wilcoxon test (covariates were not adjusted)

Our final linear regression model showed that higher breakfast frequency was associated with lower B-Pb (beta = -0.10 units of log-transformed B-Pb compared with children who did not eat breakfast regularly, p = 0.02) after controlling for covariates (Table 2). This beta, when calculate back to original scale of B-Pb, means that children eating breakfast regularly had 0.8 μg/dL (or 10%) lower B-Pb than those did not eat breakfast regularly after excluding the impacts of other factors. Other covariates including gender, age, living area, mother's education, and father's occupation were also predictors of B-Pb. While being a boy, living in a rural area, older age, and parents as professional workers were associated with higher B-Pb, children of mothers with higher education tended to have lower B-Pb. In a linear regression model of 270 children with data of calculated daily dietary intake of micronutrients (Ca, Fe, Zn, Cu, Mg, and vitamins), we did not find associations between dietary intakes of minerals and log-transformed B-Pb (data not shown). In the Logistic regression model of lead poisoning risk (Table 3), boys had 1.92 higher odds of being lead poisoning than girls, and that father's university level education reduced the odds to 0.38 times that of father with less than high school education. Compared with 3-year-old children, 4-year or 5-year old children had about 2 time odds of being lead poisoning. Regular breakfast, however, did not predict lead poisoning (B-Pb≥10 μg/dL). Blood micronutrient levels were not associated with B-Pb or the risk of lead toxicity (data not shown).

**Table 2:** Final multiple linear regression of log transformed B-Pb among children (R square = 0.14)

Variable	Parameter (beta)Estimate	StandardError	P value
Regular breakfast	-0.10	0.04	0.02

Sex (reference: boy)	-0.11	0.02	<.0001

Age (reference: 3 years)			

4	0.19	0.03	<.0001

5	0.31	0.03	<.0001

School area (reference: urban area)			

Suburban	0.02	0.02	0.45

Rural	0.07	0.03	0.02

Mother's education (reference: less than high school)			

High school	-0.07	0.03	0.01

College & University	-0.01	0.04	0.77

Father's occupation (reference: unemployed)			

General labor	0.08	0.06	0.15

Technician/Professional	0.13	0.06	0.04

Other	0.04	0.07	0.55

Mother's occupation (reference: unemployed)			

General labor	-0.05	0.05	0.36

Technician/Professional	-0.10	0.06	0.07

Other	-0.06	0.05	0.28

**Table 3:** Multiple Logistic regression analysis of elevated B-Pb

Variable	ORs	95% CI	P value
Regular breakfast			

No	Reference		

Yes	0.82	0.37~1.79	0.61

Sex			

Girl	Reference		

Boy	1.92	1.23~3.01	0.004

Age (Years)			

3	Reference		

4	1.97	1.03~3.77	0.27

5	2.33	1.24~4.38	0.03

School area			

Urban	Reference		

Suburban	0.72	0.40~1.20	0.15

Rural	1.05	0.62~1.78	0.39

Father's education			

Less than high school	Reference		

High school	0.97	0.58~1.58	0.07

College & university	0.38	0.17~0.84	0.01

Mother's education			

Less than high school	Reference		

High school	0.85	0.50~1.45	0.92

College & university	0.76	0.31~1.91	0.66

Father's occupation			

Unemployed	Reference		

General labor	1.20	0.34~4.28	0.35

Technician/professional	2.28	0.61~8.55	0.09

Other	1.75	0.42~7.31	0.61

Mother's occupation			

Unemployed	Reference		

General labor	1.65	0.54~5.04	0.21

Technician/professional	1.29	0.38~4.42	0.94

Other	1.22	0.38~3.85	0.85

ORs: odds ratios; 95% CI: 95% confidence interval

## Discussion

Previous studies showed that an empty stomach increases the absorption of lead and thus elevates B-Pb in adults, but it not clear if this effect exists in children as well. There have been no other studies directly quantifying the impact of regular breakfast consumption on lead absorption in children. In this analysis, we found that median B-Pb in those children who eat breakfast regularly was about 15% lower than that in children who do not eat breakfast regularly. This study provides important early evidence supporting the hypothesis that eating regular meals such as breakfast is associated with lower B-Pb in children. To our knowledge, this is the first human study exploring the association between breakfast frequency and B-Pb in young children. Eating frequent and regular meals (including breakfast) and snacks have been recommended by many health organizations for preventing and reducing lead poisoning in children, and these data concur. For example, the U.S. Advisory Committee on Childhood Lead Poisoning Prevention recommends that caregivers provide regular meals and snacks to young children [14].

A number of studies have shown that food in the gastrointestinal tract reduces the absorption of ingested lead in adults [7,9,10,23]. Experimental studies showed that the bioavailability of ingested lead in adults when taken with a meal was about 10 times lower than that when ingested after fasting [7]. The smaller magnitude of the influence in children (15% change) may due to relatively lower lead exposure dose in children, observational study design, and the difference in the kinetics of lead between children and adults.

The mechanisms behind the impact of fasting on gastrointestinal tract absorption of lead are not well understood. Mineral micronutrients, especially the presence of calcium and phosphate in the intestinal lumen, may play a role by competing with lead for absorption [7]. Epidemiologic studies have shown increasing dietary calcium intake is inversely associated with B-Pb in children [15,16]. Higher dietary iron intake is also associated with lower B-Pb in the U.S. children[17,18]. In the present study, however, we did not find significant associations between blood levels of these mineral micronutrients and B-Pb. We did find significantly higher blood levels of zinc in children who ate breakfast regularly, which may suggest that their dietary zinc intake was greater and perhaps competed with lead for absorption. Our analysis of dietary micronutrient intake in a subgroup of 270 children, however, did not suggest higher zinc intake in regular breakfast eaters. In previous studies, while some studies observed associations between dietary intake/supplementation (Ca, Fe, and Zn) and B-Pb [8,15-17]others did not [11,24]. In a study in the US, Gallicchio et al. did not found associations between B-Pb and daily micronutrient intakes (iron, calcium, vitamin C, or vitamin D) after controlling lead exposure and child's age [11]. A clinical trial showed that Ca supplementation did not statistically significantly change B-Pb among children 1 to 6 years of age when B-Pb were 10- 45 μg/dL [24]. There are two possible explanations for the inconsistency with this study. First, B-Pb are lower than those in previous studies. Second, children in this study are younger than the ones in previous studies.

The associations between socio-demographic factors and B-Pb found in previous studies were also observed in the present study [19,25-29]. Some of these factors including age and gender are strongly associated with B-Pb, but not modifiable. Other factors including living area and parental education and occupation might not be possible to improve solely through public health intervention. Targeting children's breakfast consumption habits is relatively more feasible at both the family and community levels.

Several limitations must be considered when interpreting the findings from the present study. We have no data on the key variable of environmental lead exposure, a factor that might explain the higher B-Pb and greater risk of toxicity in older children if cumulative lead exposure increases over time. Clearly, our findings regarding increased B-Pb with increasing age merit repeated measures over time together with measures of environmental lead. We have information of dietary intake of micronutrients on only 270 of the larger sample of 1344 children, and thus cannot be certain that these micronutrient intake levels are representative of the entire group. Parental report of regular breakfast consumption in their children may lead to bias and misclassification. A guide was provided to parents to answer this question. However, for those children primarily living with their grandparents, the information may be less accurate. We did not ask parents the number of days the child ate breakfast during the week but instead used an arbitrary definition of "at least 5 days per week" as an indication of regular breakfast consumption. This was suggested by our local partners who believed that this would reasonably distinguish children. Second, the association observed in this cross-sectional study may not represent a causal relationship. There may be a tendency for higher B-Pb's to be associated with a decreased appetite for eating breakfast. Given the data collected from this study, we cannot determine to what extent appetite affected the reported breakfast consumption. Third, the lack of association in the multivariate logistic regression analysis on lead poisoning is likely due to the limited magnitude of nutritional influence at relative low exposure level [24] and the small number of children with elevated B-Pb (7.9% for children with regular breakfast and 10.4% for children with irregular breakfast). Fourth, other potential confounders (e.g., household income and drinking water source) which were not assessed or included in the multiple regression models might contribute to the lower B-Pb observed among children who ate breakfast regularly. Finally, given the impact of fasting on lead absorption, regular breakfast is expected to modify the associations between external exposure levels and B-Pb which we cannot examine in this study due to lack of external exposure data.

Our findings, if replicated in future studies, have three practical implications. First, the importance of breakfast consumption as a strategy to increase meal frequency is suggested. Second, our data suggest that the risk of lead toxicity and higher B-Pb increases over time, a finding that suggests consideration of environmental routes of lead exposure. Third, we found that parental characteristics are major determinants of children's breakfast frequency. Children from families with higher parental education levels and technicians/professional workers are more likely to have regular breakfast. This indicates that parents play important role in increasing children's breakfast frequency which may in turn reduce B-Pb. Promotion of parents' and child care givers' awareness of the importance of breakfast as a proactive protective factor in reducing childhood B-Pb should become a part of all lead prevention programs. Because both lead exposure[1-6,30-33] and malnutrition[34-37]are related to children's negative cognitive and behavioral outcomes, promotion of regular breakfast in children can potentially enhance both physical and mental health well-being.

## Conclusions

This study provided epidemiological evidence that increasing breakfast frequency could reduce B-Pb in young children. Parental or caregivers' characteristics including education and occupation are major determinants of breakfast frequency, indicating that improving their knowledge about nutrition and B-Pb might help to prevent lead poisoning.

## List of Abbreviations

B-Pb: blood lead Levels; CDC: Centers for Disease Control and Prevention; QC: Quality Control; Fe: iron; Cu: copper; Zn: zinc; Ca: calcium; Mg: magnesium

## Competing interests

The authors declare that they have no competing interests.

## Authors' contributions

JL conducted data collection, data analysis, data interpretation, and manuscript preparation. LM, CC, and JP conducted data interpretation and manuscript preparation. CY and XS conducted blood lead analysis. All authors read and approved the final manuscript.
